# SGLT-2 inhibitors in prevention of chemotherapy-induced cardiotoxicity: systematic review and meta-analysis

**DOI:** 10.1093/ehjopen/oeag012

**Published:** 2026-01-31

**Authors:** Soomal Rafique, Michael Buhnerkempe, Yansoun Elmasry, Abhishek Kulkarni, Krishna Rao, Mukul Bhattarai

**Affiliations:** Department of Internal Medicine, SIU School of Medicine, Springfield, IL 62702, USA; Center for Clinical Research, SIU School of Medicine, Springfield, IL 62702, USA; Cardiology Division, Department of Internal Medicine, SIU School of Medicine, Springfield, IL 62702, USA; Cardiology Division, Department of Internal Medicine, SIU School of Medicine, Springfield, IL 62702, USA; Hematology-Oncology Division, Department of Internal Medicine, SIU School of Medicine, Springfield, IL 62702, USA; Cardiology Division, Department of Internal Medicine, SIU School of Medicine, Springfield, IL 62702, USA

**Keywords:** Chemotherapy, Cardiac dysfunction, Heart failure, Sodium-glucose cotransporter-2 inhibitors, Meta-analysis

## Abstract

**Aims:**

Chemotherapy is associated with significant cardiotoxicity. Although other guideline directed medications for heart failure are effective in managing or preventing these cardiotoxic effects, the potential role of sodium-glucose cotransporter-2 (SGLT2) inhibitors remain incompletely understood.

**Objectives:**

This study aims to systematically review high-quality studies to evaluate the cardiovascular outcomes associated with SGLT2 inhibitor use in cancer patients undergoing chemotherapy.

**Methods and results:**

We conducted a meta-analysis of cohort studies comparing cardiovascular outcomes between cancer patients receiving SGLT2 inhibitors and those not receiving SGLT2 inhibitors. Cochrane Central Register, clinicaltrials.gov, PubMed, Embase, and Google Scholar were searched from inception to May 2025. Primary outcome was all-cause mortality. Secondary outcomes included cardiac events, and cardiac dysfunction. We used fixed and random effect binomial meta-analysis fit using the Mantel–Haenszel method for each outcome of interest. All analyses were conducted using the ‘meta’ package in R Statistical Software. Eleven cohort studies comprising 2 689 260 patients were included, of whom 29 958 received SGLT2 inhibitors. SGLT2 inhibitor use was significantly associated with reduced all-cause mortality (OR 0.27, 95% CI 0.25–0.28), cardiac events (OR 0.49, 95% CI 0.49–0.53), cardiac dysfunction (OR 0.62, 95% CI 0.56–0.69), and heart failure hospitalizations (HFH; OR 0.67, 95% CI 0.61–0.75) compared to non-use.

**Conclusion:**

SGLT2 inhibitors demonstrate robust cardioprotective effects in cancer patients receiving chemotherapy, significantly reducing mortality, HFH, and major cardiac events. These findings support the integration of SGLT2 inhibitors into cardio-oncology strategies, particularly for patients at high risk of chemotherapy-induced cardiotoxicity.

## Introduction

Cancer therapy-related cardiac dysfunction (CTRCD) poses a significant risk of morbidity and mortality in patients receiving cardiotoxic cancer treatments.^1^ Despite the effectiveness of traditional medications such as beta-blockers, angiotensin-converting enzyme inhibitors, angiotensin receptor blockers, and angiotensin receptor-neprilysin inhibitors in managing or preventing these cardiotoxic effects,^2–4^ the potential role of sodium-glucose cotransporter-2 (SGLT2) inhibitors is still not well understood.^5^ In the current era, where the incidence of cancer continues to rise and treatment regimens become increasingly complex, it is crucial to explore all available options for protecting cardiovascular health in this vulnerable population. This study aims to systematically review high-quality cohort studies to evaluate the cardiovascular outcomes associated with SGLT2 inhibitor use in cancer patients undergoing several chemotherapy regimens. SGLT2 inhibitors may provide noteworthy benefits for heart health by promoting cell survival and reducing cell death.^6^ Research, such as that by Dukta et al. in 2022, highlights how these inhibitors can diminish inflammation by targeting reactive oxygen species and inflammatory cytokines.^7^ Given their mechanism of action, SGLT2 inhibitors may play a vital role in alleviating cardiac toxicity that arises from chemotherapy,^8,9^ making them an important area of study in the context of CTRCD.

In addition to their cardioprotective properties, SGLT2 inhibitors could enhance cancer treatment outcomes. They work by decreasing glucose uptake in cancer cells, limiting systemic glucose availability, and modulating various signalling pathways while regulating the expression of specific genes and proteins.^7,8^ These multifaceted actions might produce a synergistic effect with chemotherapy, potentially improving overall treatment efficacy. As the landscape of cancer treatment evolves, it is essential to investigate how SGLT2 inhibitors can be integrated into patient care to optimize both cardiovascular and cancer treatment outcomes.

## Methods

A comprehensive search of electronic databases was conducted to identify studies evaluating the effectiveness of SGLT2 inhibitors in preventing cardiac dysfunction among patients treated with cardiotoxic cancer therapies and subsequently diagnosed with cardiomyopathy.

### Search strategy and selection criteria

Databases searched included the Cochrane Central Register of Controlled Trials, ClinicalTrials.gov, PubMed, Embase, and Google Scholar, from inception to May 2025. The search incorporated keywords and MeSH terms related to SGLT2 inhibitors, cardiotoxic agents (e.g. doxorubicin, anthracyclines), cancer, cardiotoxicity, cardiovascular outcomes, and mortality. Final study selection was made by author consensus, following PRISMA guidelines.^10^ IRB approval was not required for this study as it was limited to the analysis of data from previously published studies and does not involve the collection or use of new identifiable patient information.

### Inclusion criteria

Eligible studies were randomized controlled trials or observational studies involving adults (≥18 years) undergoing cardiotoxic chemotherapy, immunotherapy, or radiotherapy. Studies had to compare cardiovascular outcomes, such as all-cause mortality, heart failure hospitalizations (HFH), or cardiac dysfunction between SGLT2 inhibitor users and non-users. Comparisons included drug vs. placebo, drug vs. drug, or combination regimens. Studies were included regardless of cancer type or diabetes status, provided relevant cardiovascular endpoints were reported.

### Exclusion criteria

Excluded were preclinical studies, case reports, case series, reviews, editorials, letters, studies without a comparator group, those lacking cardiovascular outcomes, paediatric studies (<18 years), non-English publications, overlapping datasets (only the most complete or recent version retained), and studies with insufficient outcome data.

### Study selection and characteristics

**Figure 1:**
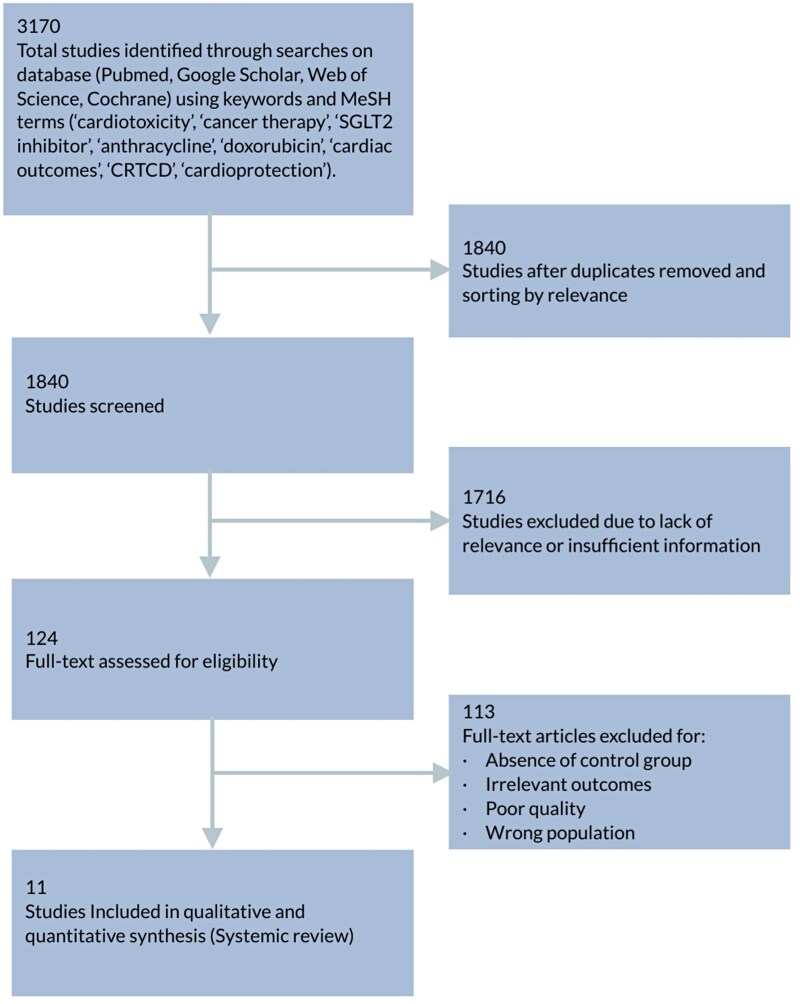
PRISMA flow diagram of study selection.

As shown in *Figure 1*, a total of 3170 records were identified. After removing duplicates, 1840 studies were screened by title and abstract. Of these, 1716 were excluded due to irrelevance or insufficient data. Full-text review was conducted on 124 articles, with 113 excluded based on predefined criteria. Eleven studies were included in the final systematic review and meta-analysis. The ongoing EMPACT Trial (NCT05271162), although relevant, was excluded due to lack of published data.

### Outcomes

The primary outcome was all-cause mortality. Secondary outcomes included heart-failure hospitalization, composite cardiac events, and cardiac dysfunction.

### Outcomes definitions

#### All-cause mortality

Defined as death from any cause. Mortality ascertainment was based on the method used in each original study, including adjudicated death records, national mortality databases, clinical documentation, or ICD-coded administrative data.

#### Heart-failure hospitalization (HFH)

Defined as hospital admission for acute decompensated heart failure or new-onset heart failure in patients without prior cardiomyopathy. Depending on the study, HFH identification relied on clinical diagnosis, adjudicated HF events, or ICD-10 coding. Some studies (e.g. Avula et al.) evaluated acute HF exacerbations among patients with established cardiomyopathy, whereas others (e.g. Bhatti et al.) focused on incident HF in patients with no prior HF history.

#### Composite cardiac events

Accepted as defined in each original publication. These typically included heart-failure hospitalization, myocardial infarction, ischaemic stroke, new arrhythmias, cardiovascular hospitalization, or death. For example, Hwang et al. included HFH, MI, stroke, and death, whereas Chiang et al. defined events as HFH and all-cause mortality.

#### Cardiac dysfunction

Defined according to each study’s operational criteria, most commonly representing new-onset heart failure, incident cardiomyopathy, or newly identified left-ventricular systolic dysfunction based on clinical diagnosis, imaging interpretation, adjudicated events, or ICD coding.

### Data extraction and quality assessment

Two reviewers independently extracted data from the included studies using a pre-designed excel sheet. The extracted information included the first author, publication year, country, study period, study type, number of patients, mean age, type of cancer and chemotherapy, study outcomes, and average follow-up years. Any discrepancies were resolved by discussion with a third reviewer.

### Statistical analysis and data synthesis

To determine the impact of SGLT2 inhibitor use on our outcomes of interest, we used a fixed-effect binomial meta-analysis fit using the Mantel–Haenszel method for each outcome of interest. Heterogeneity across studies was assessed using the *I*^2^ statistic, where values over 50% potentially indicate substantial heterogeneity.^11^ Where significant heterogeneity in study outcomes existed, we also used a random-effects meta-analysis fit using the Mantel–Haenszel method. Publication bias was assessed visually using funnel plots due to the small number of studies included. All analyses were conducted using the ‘meta’ package^12^ in R Statistical Software.^13^

## Results

A total of 11 studies were included in the analysis.^5,14–23^ Six studies were conducted in the United States,^5,14,15,18,22,23^ two in Taiwan,^19,21^ and one each in Canada,^17^ South Korea,^20^ and Israel.^16^ The study durations ranged from 1.5 to 9.1 years. The total patient population comprised 2 689 260 individuals, with 29 958 in the SGLT2 inhibitor group and 2 659 302 in the control group. The mean age of participants ranged from 60 to 74.8 years. All patients had type 2 diabetes, except for one study that included 10% nondiabetic individuals.^14^ Chemotherapy regimens included anthracyclines, alkylating agents, antimetabolites, platinum-based therapies, tyrosine kinase inhibitors, and immune checkpoint inhibitors. Three studies focused exclusively on cancer patients treated with anthracyclines,^14,17,22^ although cumulative anthracycline doses were not reported. Most studies encompassed multiple cancer types, except for two that focused on nonsmall cell lung cancer^18^ and hepatoma.^22^ The average follow-up duration ranged from 1 to 2.33 years. *Tables 1* and *2* summarize the characteristics of the included studies.

**Table 1:** Study characteristics

Study name	Year	Country	Study type	Study period (years)	Follow-up (years)	Type of malignancy	Chemotherapy type	Type of SGLT2-I	Cumulative dose of anthracycline	Outcomes	Propensity score matched (yes/no)
**Fath et al** ^14^	2024	US	Retrospective	8	2	Haematological, breast, GI cancer	Anthracycline (Doxorubicin)	Empagliflozin 57%, Canagliflozin 31%, Dapagliflozin 26%	NA	New-onset HF, arrhythmia, MI, all-cause mortality, hospitalization	Yes
**Avula et al** ^5^	2024	US	Retrospective	7.4	2	MDS, lymphoma, breast cancer	Fluorouracil, Cyclophosphamide, Bevacizumab, Doxorubicin	Empagliflozin, Dapagliflozin or Canagliflozin	NA	HF exacerbation, all-cause mortality, all-cause hospitalizations, ED visits, AF	Yes
**Bhatti et al** ^15^	2024	US	Retrospective	9.1	1	Lymphoma, breast and, gastrointestinal cancers	Anthracycline, monoclonal antibodies, antimetabolites.	Empagliflozin, Dapagliflozin or Canagliflozin	NA		Yes
**Perelman et al** ^23^	2024	Israel	Retrospective	7	2.33	NSCLC, RCC, HCC and others	Immune checkpoint inhibitors	Empagliflozin 83%, Dapagliflozin 17%	NA	All-cause mortality, MACE, arrhythmia	Yes
**Abdel-Qadir et al** ^21^	2023	Canada	Retrospective, multicenter	4	1.5	Breast cancer, lymphoma	Anthracycline (Doxorubicin, Epirubicin)	Dapagliflozin, Empagliflozin, Canagliflozin	NA	HF hospitalization, new HF diagnosis, CVD hospitalization, all cause death	Yes
**Luo et al** ^16^	2023	US	Retrospective	3	1.5	Noncell lung cancer	Not specified	Canagliflozin, Dapagliflozin, Empagliflozin, and Ertugliflozin	NA	All-cause mortality	Yes
**Huang et al** ^19^	2023	Taiwan	Retrospective	5	4.5	All	Not specified	Not specified	NA	Cancer-specific and all-cause mortality	Yes
**Hwang et al** ^22^	2023	South Korea	Retrospective	7	N/A	Breast, lymphoma, GU, other cancers	Doxorubicin, Epirubicin, HER2 inhibitors, VEGF targeting agents	Not specified	NA	HFH, AMI, ischemic stroke, death	Yes
**Chiang et al** ^20^	2022	Taiwan	Retrospective	2	1.6	All, mostly gastrointestinal and genitourinary cancers	Anthracyclines (8%), tyrosine kinase inhibitors (4%), Plant alkaloids, Platinum HER2 inhibitors, VEGF inhibitors, Immune checkpoint inhibitors	Empagliflozin (49%), Dapagliflozin (38%)	NA	HFH, all-cause mortality, serious adverse events with SGLT2i.	Yes
**Gongora et al** ^17^	2022	US	Retrospective	1.5	1.5	Lymphoma, breast cancer, and various others	Anthracycline (Doxorubicin)	Empagliflozin 50%, Canagliflozin 34%, Dapagliflozin 16%	NA	HF admission, new cardiomyopathy, arrhythmia, HF incidence, mortality	Yes
**Hendryx et al** ^18^	2022	US	Retrospective	3	1.7	Hepatocellular carcinoma	Not specified	Canagliflozin, Dapagliflozin, Empagliflozin, and Ertugliflozin	NA	All-cause mortality	Yes

AF, atrial fibrillation; MI, myocardial infarction; AKI, acute kidney injury; AMI, acute myocardial infarction; CM, cardiomyopathy; CVD, cardiovascular disease; DKA, diabetic ketoacidosis; HER, human epidermal growth factor receptor; HF, heart failure; HFH, heart failure hospitalization; MACE, major adverse cardiovascular event; NSCLC, nonsmall cell lung cancer; RCC, renal cell carcinoma; UTI, urinary tract infection; VEGF, vascular endothelial growth factor.

**Table 2:** Baseline characteristics

Study name	Total number of patients (*n*)	SGLT2-I (*n*)	Non-SGLT2-I group	Number of diabetes (%)	Mean age (yrs.)	Inclusion criteria	Exclusion criteria	Outcome/results	Definition of LV dysfunction
**Fath et al** ^14^	91 103	724	78 350	90	62.5	Age >18, diagnosed with cancer, received anthracyclines, no prior HF	No pre-existing heart failure	Lower new-onset HF and arrhythmia in SGLT2i group, similar mortality and hospitalization rates	NA
**Avula et al** ^5^	1280	640	640	100	67.6	Age >18, DM2, cardiotoxic cancer therapies, diagnosed with cardiomyopathy/HF	Diagnosis of ACS, CABG or PCI after starting antineoplastic therapy	SGLT2i associated with reduced risk of HF exacerbation and mortality	NA
**Bhatti et al** ^15^	95 203	9403	85 800	100	65.5	Age ≥18, with T2DM, cancer, exposure to cardiotoxic therapies, and no prior documented history of cardiomyopathy or heart failure	Age <18, nondiabetic, not on diabetic treatment, prior heart failure	SGLT2i administration was associated with a significantly decreased risk of developing CTRCD in patients with T2DM and cancer	N/A
**Perelman et al** ^23^	119	24	95	100	71	Age >18, DM2, treated with ICIs therapy	Age <18	SGLT2i linked to lower all-cause mortality in patients with DM2 and cancer treated with ICIs	NA
**Abdel-Qadir et al** ^21^	933	99	834	100	68-76	Age >18, treated DM2,, received anthracyclines, no prior HF	Age <18, nondiabetic, not on diabetic treatment, prior heart failure	No HF hospitalization in SGLT2i group, no significant difference in mortality	NA
**Luo et al** ^16^	24 915	531	24 384	100	72.5	Age ≥ 65, newly diagnosed NSCLC, DM2	Age <65, without DM2 or NSCLC	Improved overall survival of NSCLC patients with pre-existing diabetes	N/A
**Huang et al** ^19^	50 133	16 711	33 422	100	62	Age > 20, DM2, receipt of at least one type of antidiabetic medication, and presence of cancer without metastasis	History of cancer before diagnosis of T2DM and that of synchronous or metachronous double cancers.	SGLT2is increase overall survival and cancer-specific survival in patients with cancer in a dose-dependent manner	N/A
**Hwang et a**l^22^	3116	779	2337	100	61	Age ≥18, newly diagnosed with cancer and underwent AC-containing chemotherapy	Patients with metastasis, preexisting significant cardiac diseases, diabetes, insulin use, stroke, etc.	SGLT2i associated with improved outcomes, including HF hospitalization, AMI, stroke, and mortality	NA
**Chiang et al** ^20^	1756	878	878	100	65	Age >18, DM2, cancer diagnosis	missing data or insufficient visits, age <18	SGLT2i associated with a lower rate of incident HF and prolonged overall survival in cancer patients with DM2	NA
**Gongora et al** ^17^	128	32	96	100%	60	Age >18, cancer diagnosis, received anthracyclines, DM2	Pre-existing heart failure, significant arrhythmias, severe systemic diseases	Lower incidence of heart failure, better overall survival, lower sepsis/neutropenic fever in SGLT2i group (*P* < 0.001)	NA
**Hendryx et a**l^18^	274	137	137	100	74.8	Age ≥ 66, newly diagnosed HCC, DM2	Age <66, without DM2 or HCC	Improved overall survival of HCC patients with pre-existing diabetes	N/A

DM2, type 2 diabetes mellitus; HCC, hepatocellular carcinoma; CTRCD, cancer therapy-related cardiac dysfunction.

**Figure 2:**
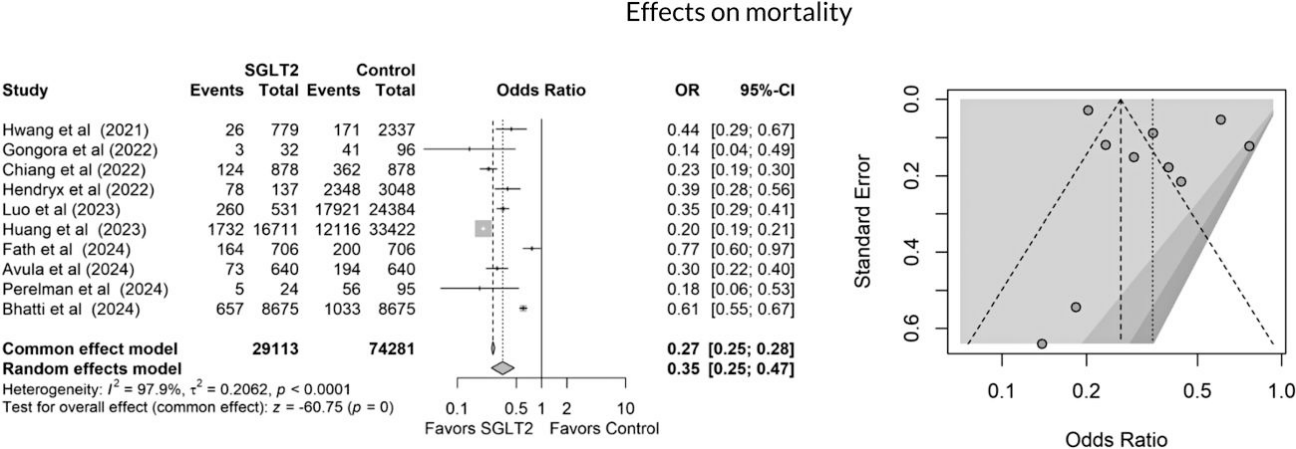
Forest and funnel plots showing effects of SGLT2 inhibitors on mortality.

**Figure 3:**
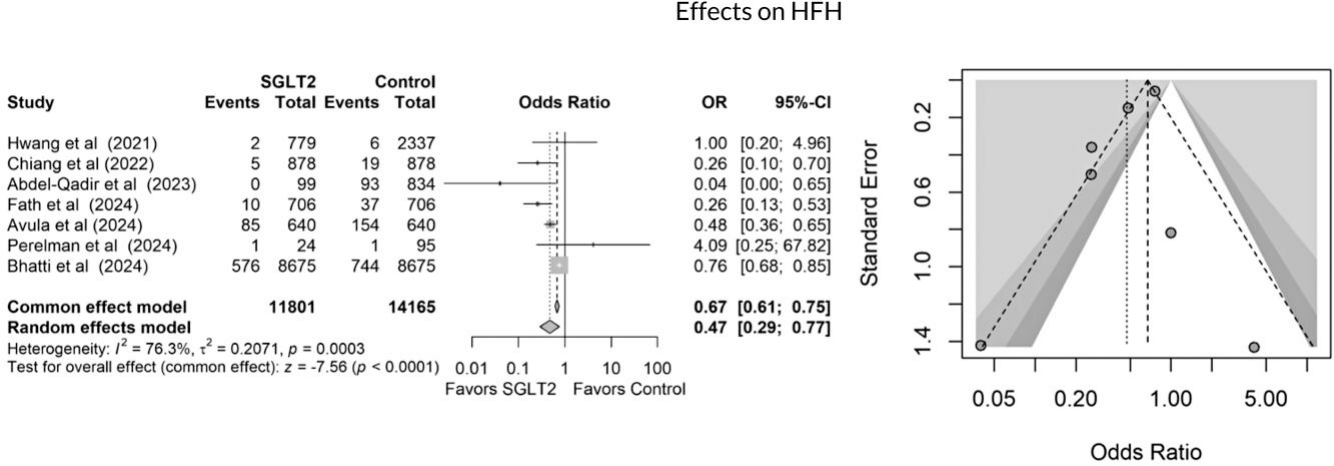
Forest and funnel plots showing effects of SGLT2 inhibitors on heart failure hospitalizations (HFH).

**Figure 4:**
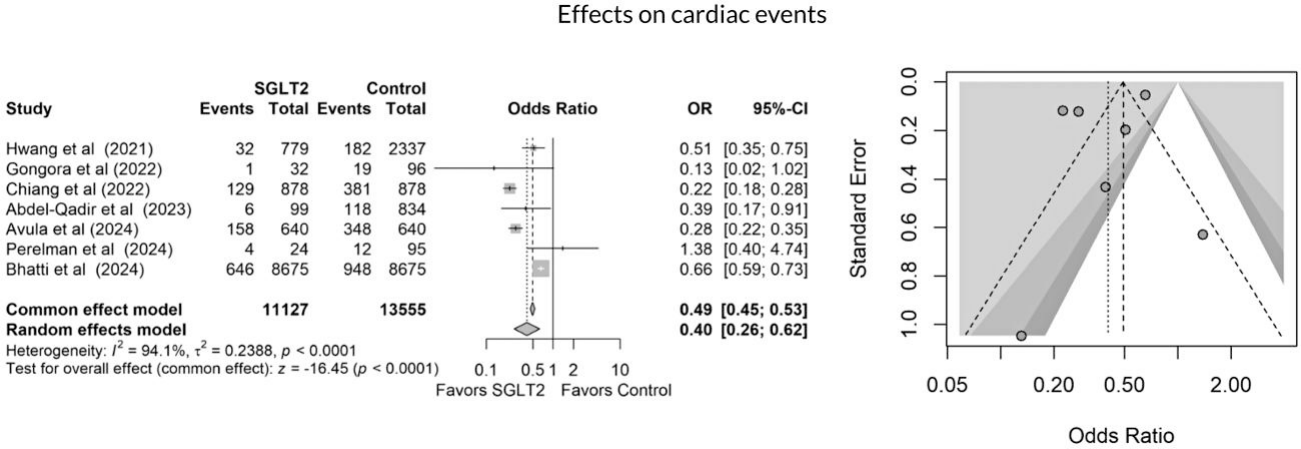
Forest and funnel plots showing effects of SGLT2 inhibitors on cardiac events.

**Figure 5:**
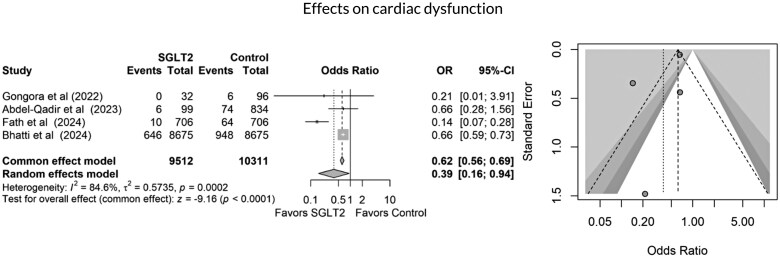
Forest and funnel plots showing effects of SGLT2 inhibitors on cardiac dysfunction.

All studies included in the quantitative meta-analysis reported all-cause mortality as an outcome. As shown in *Figure 2*, SGLT2 inhibitor use was associated with a significantly lower risk of mortality compared to non-use (3122 of 11 801 in the SGLT2 inhibitor group vs. 34 442 of 74 281 in the control group; OR 0.27, 95% CI 0.25–0.28, *P* < 0.0001). This corresponds to an absolute risk reduction (ARR) of 19.9% and a number needed to treat (NNT) of 5 to prevent one death. SGLT2 inhibitor use was also associated with a 43% relative reduction in mortality risk. However, high heterogeneity was observed in this pooled result (*I*² = 97.9%). Seven studies reported HFH. SGLT2 inhibitor use was associated with a significantly lower risk of hospitalization due to heart failure (679 of 11 801 vs. 1054 of 14,165, OR 0.67, 95% CI 0.61–0.75, *P* < 0.0003), as shown in *Figure 3*. Seven studies demonstrated a reduction in all cardiac events in the SGLT2 inhibitor group compared to the control group (976 of 11 127 vs. 2008 of 13,555, OR 0.49, 95% CI 0.49–0.53, *P* = 0.0001, I² = 94.1%), as illustrated in *Figure 4*. Finally, cardiac dysfunction was reported in four studies (*Figure 5*). SGLT2 inhibitor use was associated with a significantly lower incidence of cardiac dysfunction (662 of 9512 vs. 1092 of 10,311, OR 0.62, 95% CI 0.56–0.69, *P* < 0.0002). The survival and cardioprotective benefits of SGLT2 inhibitors in chemotherapy-induced cardiotoxicity are likely multifactorial, involving improved diabetes control, favourable metabolic effects, and modulation of cancer biology and systemic inflammation. These noncardiac mechanisms complement direct cardiac protection and contribute to the overall improved outcomes observed in cardio-oncology populations.

## Discussion

This meta-analysis demonstrates that SGLT2 inhibitor use in cancer patients undergoing cardiotoxic chemotherapy is associated with a significant reduction in all-cause mortality, HFH, cardiac dysfunction, and overall cardiac events. The observed ARR of 19.9% in all-cause mortality and NNT of 5 underscores the substantial clinical benefit of SGLT2 inhibitors in this high-risk population. These findings are particularly important given the growing population of cancer survivors at increased risk for cardiovascular complications due to chemotherapeutic regimens. The cardioprotective effects observed across diverse cancer types and treatment protocols, including anthracycline-based regimens, suggest a potential role for SGLT2 inhibitors as a therapeutic strategy to mitigate CTRCD, regardless of diabetic status. The cardioprotective mechanisms of SGLT2 inhibitors are multifactorial and well-supported by evidence from major cardiovascular outcome trials such as DECLARE-TIMI 58^24^ and the CANVAS program.^25^ In recent years, their potential in mitigating CTRCD has gained increasing attention. Meta-analyses by Mir et al.^26^ and Kuo et al.^27^ have compiled data supporting the cardioprotective role of SGLT2 inhibitors in patients undergoing chemotherapy. Mechanistically, these agents are known to reduce oxidative stress, systemic inflammation, and cardiomyocyte apoptosis; key contributors to CTRCD.^28^ They also enhance myocardial metabolism and improve haemodynamic parameters, which are crucial in preserving cardiac function.^7–9^ These protective pathways are especially pertinent in the context of commonly used cardiotoxic agents, such as anthracyclines,^29^ trastuzumab, and tyrosine kinase inhibitors.^28^ By attenuating these adverse cardiac effects, SGLT2 inhibitors not only preserve cardiac function but may also support uninterrupted completion of cancer therapy, thereby optimizing both oncologic and cardiovascular outcomes.^30^ A 2025 meta-analysis by Bhalraam et al. reported that SGLT2 inhibitors substantially improve heart-failure outcomes in cancer patients and survivors. Across 13 studies involving 88 273 participants, SGLT2 inhibitor use was linked to a 51% reduction in heart-failure hospitalizations and a 71% decrease in new heart-failure diagnoses. Notably, in breast-cancer cohorts where at least half of patients had received anthracyclines, multivariate meta-regression revealed a striking 99% reduction in hospitalization risk compared with studies with lower anthracycline exposure.^30^ A recent editorial highlights evidence showing that SGLT2 inhibitors substantially reduce all-cause mortality and HFH compared with other diabetes medications. In addition to these cardioprotective effects, the authors note emerging preclinical and clinical findings suggesting that SGLT2 inhibitors may also exert anticancer benefits by limiting tumour glucose uptake, activating metabolic-regulatory pathways such as AMPK, inhibiting proliferative signalling pathways including PI3K/AKT/mTOR and Hippo, promoting tumour-suppressor activity, and reducing inflammation, oxidative stress, and cancer cell growth and migration.^31^ Furthermore, EMPACARD-PILOT trial, a prospective case-control study, also demonstrated a significant reduction in CTRCD among high-risk breast cancer patients receiving anthracycline-based chemotherapy who were treated with empagliflozin.^32^ Despite their known side-effect profile, emerging evidence supports the use of SGLT2 inhibitors in cancer patients, including those with renal or bladder cancer, as their cardiorenal benefits appear to extend to oncology populations. Rates of urinary tract infection are not significantly higher than with other antidiabetic agents, though caution is appropriate in patients with recurrent or severe infections. Euglycaemic diabetic ketoacidosis remains a rare but serious adverse effect (<0.1% incidence), underscoring the need for careful monitoring.^33,34^ This meta-analysis offers several strengths, including a large, pooled sample size and inclusion of diverse cancer populations undergoing a range of cardiotoxic treatments. The consistent reduction in mortality and cardiac events across multiple outcomes reinforces the robustness of the findings. Several limitations should be noted. Most included studies were observational, raising the possibility of residual confounding. There was substantial heterogeneity in cancer types, chemotherapy regimens, baseline cardiovascular risk, and follow-up duration. Reporting of cumulative chemotherapy dose, timing of SGLT2 inhibitor initiation, and medication adherence was incomplete in many cohorts. Because several studies predated the European Society of Cardiology (ESC) 2022 Cardio-Oncology Guidelines—which define CTRCD as a ≥10% absolute decline in left ventricular ejection fraction (LVEF) to <50% or a >15% reduction in global longitudinal strain (GLS),^35^ none applied this standardized criterion, contributing to outcome heterogeneity. Additionally, one study (Fath et al.) did not show a mortality benefit, which may relate to differences in population characteristics, follow-up duration, or the proportion of diabetic vs. nondiabetic patients. Cardiotoxicity outcomes were inconsistently reported, and key clinical details such as cumulative anthracycline dose, timing and duration of SGLT2 inhibitor therapy, and potential differences among SGLT2 inhibitor subtypes were not adequately described, further limiting comparability across cohorts.

Despite these limitations, the magnitude of observed benefit suggests a compelling rationale for prospective, randomized controlled trials to evaluate the role of SGLT2 inhibitors as cardioprotective agents in oncology settings. If confirmed, these agents may become integral to cardio-oncology practice, particularly in patients at high risk for CTRCD.

Future research should prioritize prospective, randomized controlled trials specifically designed to evaluate the cardioprotective effects of SGLT2 inhibitors in cancer patients, including those without diabetes. Stratification by cancer type, chemotherapy regimen, and baseline cardiovascular risk will be essential to identify subgroups that derive the greatest benefit. Biomarker-guided strategies and cardiac imaging endpoints could enhance early detection of subclinical cardiotoxicity and clarify mechanisms of benefit. From a clinical perspective, the integration of SGLT2 inhibitors into cardio-oncology care pathways warrants careful consideration, especially given their favourable safety profile and established benefits in heart failure and diabetes. Early initiation of SGLT2 inhibitors in high-risk cancer patients may represent a proactive strategy to preserve cardiac function and allow uninterrupted cancer therapy. Collaborative efforts between oncologists and cardiologists will be crucial to translating these findings into practice and optimizing long-term outcomes for cancer survivors.

In conclusion, this systematic review and meta-analysis demonstrate that SGLT2 inhibitors are associated with substantial reductions in mortality, HFH, and cardiac dysfunction in cancer patients receiving cardiotoxic chemotherapy. These findings highlight the emerging role of SGLT2 inhibitors as cardioprotective agents in oncology. While encouraging, confirmation through large-scale randomized trials is needed to inform clinical guidelines. As the field of cardio-oncology evolves, integrating SGLT2 inhibitors into preventive strategies may significantly improve cardiovascular and oncologic outcomes in this vulnerable population. Importantly, these benefits may be especially relevant for patients classified as high or very-high risk for CTRCD according to the ESC HFA-ICOS baseline risk assessment tool,^35^ such as individuals receiving highly cardiotoxic regimens (e.g. high-dose anthracyclines or sequential anthracycline–trastuzumab therapy) or those with significant cardiovascular comorbidity—who represent the population most vulnerable to treatment-related cardiotoxicity.

## Data Availability

Data included in manuscript or supplements.
